# Role of lipid apheresis in changing times

**DOI:** 10.1007/s11789-012-0049-3

**Published:** 2012-05-03

**Authors:** Peter Schuff-Werner, Sebastian Fenger, Peter Kohlschein

**Affiliations:** Institut für klinische Chemie und Laboratoriumsmedizin, Universitätsmedizin Rostock, Ernst-Heydemann-Straße 6, 18057 Rostock, Germany

**Keywords:** History of LDL-apheresis, Efficacy of LDL-lowering, Longterm treatment, Clinical effects and patient’s outcome after LDL-apheresis, Future lipid-lowering strategies

## Abstract

During the last decades, LDL-apheresis was established as an extracorporeal treatment option for patients with severe heterozygous or homozygous familial hypercholesterolemia (FH) that is resistant to conventional treatment strategies such as diet, drugs, and changes in lifestyle. Nearly half a century ago, the first LDL-apheresis treatment was performed by plasma exchange in a child with homozygous FH.

At the beginning of the 1970s, the clinical advantage of regular extracorporeal LDL-elimination was demonstrated in siblings suffering from homozygous FH. These findings encouraged researchers especially from Germany and Japan to develop extracorporeal devices to selectively eliminate LDL-cholesterol in the 1980s.

Although the selectivity of the currently available LDL-apheresis devices is different, the efficacy of LDL-elimination during a single treatment is rather similar and ranges between 55 and 65 % of the pretreatment LDL plasma concentration.

In the 1990s, the patients regularly treated by extracorporeal LDL-elimination, diet, and drugs were included in regression studies assessed by angiography. It was shown that the combined treatment with LDL-apheresis, diet, and drugs resulted in less progression of coronary lesions than drugs and/or diet alone. However, although a tendency was evident, results did not reach criteria for significance.

During the last decade, apheresis registries were established to collect data on efficiency, safety, and clinical outcome of regular long-term LDL-apheresis. The evaluation of registry data will certainly permit further insights in the therapeutic benefit of this expensive and time-consuming therapeutic approach.

Furthermore, the future of LDL-apheresis will depend upon the availability of highly efficient new drugs and molecular genetic approaches such as RNA silencing of the apoB gene, whereas the liver transplantation and gene therapy of the LDL-receptor deficiency will not replace LDL-apheresis in severe familial hypercholesterolemia in the near future.

## Hypercholesterolemia refractory to conventional therapy

Familial hypercholesterolemia (FH) is one of the most common inherited metabolic diseases, caused in most cases by different mutations of the LDL receptor. Previously, this disease in its homozygous form resulted in the early childhood to atherosclerotic vascular changes, especially involving coronary arteries and the aorta with consecutive severe aortic stenosis [11]. The life expectancy was rarely longer than 20 years; a causal therapy was not available for a long time. Because of the receptor defect, the approach to therapy with diet and the first generation of lipid-lowering medication was ineffective in as much as the desired increased receptor expression in these patients is not sufficient to lower the highly elevated LDL cholesterol serum levels. This dilemma was the reason to take alternative treatment strategies into consideration based on an increasing body of evidence that there is a strong relationship, both epidemiologically and pathophysiologically, between elevated serum cholesterol levels and progressing atherosclerotic lesions.

## Unselective plasma exchange as a precursor of LDL apheresis (before 1980)

The first elimination of plasma (plasmapheresis) with reinfusion of blood cells was performed in 1914 by Abel et al. in dogs [1]. In patients with Waldenstroem’s macroglobulinemia along with hyperviscosity syndrome, plasma exchange treatment could be successfully used for the first time in 1960 [38].

For the treatment of FH, the concept of a plasma exchange was introduced in the 1960s by de Gennes [7] treating a young girl with homozygous familial hypercholesterolemia for the first time. The case report of this first successful treatment therefore marks the beginning of LDL apheresis as a therapeutic option for patients with FH.

In the following years, the nonselective LDL removal by plasma exchange in young FH patients was performed by Thompson. He demonstrated for the first time the clinical efficacy of this treatment in a very impressive way. Insofar, the patients treated by plasma exchange survived impressively than their siblings who were treated only by diet and the available drugs at that time [50].

## Development of selective LDL-apheresis methods (1981–1993)

To avoid risks for patient associated with pure plasma exchange and with the exchange of plasma versus human albumin-salt solutions, various methods for selective elimination of atherogenic lipoproteins were developed and clinically tested since the early 1980s (Table 1).

**Table 1:** Overview of the various lipid-apheresis procedures and the underlying methodological principles and the reported LDL-lowering efficacy

Year of introduction	Procedure	Principle	LDL-reduction (%)	Reference
1966	Plasmapheresis	LDL-elimination by plasma exchange		[7]
1980	Heparin-adsorption	LDL-adsorption (Plasma)		[21]
1981	Immunoadsorption	LDL-adsorption (Plasma)	35–56	[41]
1983	Differential filtration	LDL-filtration (Plasma)	56–62	[51]
1984	Dextran sulfate adsorption	LDL-adsorption (Plasma)	49–75	[55]
1985	Thermofiltration	LDL-filtration (Plasma)	61	[26]
1986	Heparin-induced extracorporeal LDL-precipitation (H.E.L.P.)	LDL-precipitation (Plasma)	55–61	[3]
1992	Direct adsorption of lipids (DALI)	LDL-adsorption (whole blood)	53–76	[5]

The immunoadsorption of apolipoprotein (Apo) B is based on its binding to immobilized anti-apoB antibodies [41]. The immunoapheresis is thus a very specific LDL apheresis method, eliminating all ApoB containing lipoprotein particles such as LDL, Lp(a), and some VLDL [32]. Later, this method was modified by the use of monoclonal antibodies against ApoB or specific antibodies against Lp(a), respectively [28].

Around the same time, a molecular-sieving procedure that separates plasma proteins of high molecular size from plasma, including LDL, was introduced as an extracorporeal LDL-eliminating device. The disadvantage of this method was that beside LDL the plasma concentrations of some other proteins, such as albumin and immunoglobulin M, as well as the antiatherogenic HDL were significantly lowered. Therefore, this lipid filtration procedure is far less selective than the immunoadsorption procedure, even if due to improved filtering technologies, the actual device reduces albumin and HDL to a less extent than IgM [51,10].

In the mid 1980s, chemically defined ApoB-adsorbing polyanions such as dextran sulfate or heparin were covalently bound to column matrix in order to eliminate LDL from plasma.

Immobilized heparin bridges via disulphide moieties the ApoB bearing LDL, but it is disadvantageous that all other heparin-binding proteins are also removed, mainly clotting factors [21]. Therefore, this method was not established for routine clinical use whereas the so-called dextran sulfate adsorption became one of the world’s most common LDL apheresis methods [55].

Clinical chemists and laboratory physicians of the Goettingen University Hospital, however, hold to the idea of using heparin for extracorporeal LDL-elimination: For lipid analysis, they used the pH-dependant property of heparin, to precipitate lipoproteins in vitro [53]. This principle was then brought to clinical application by Victor W. Armstrong in collaboration with the B. Braun Melsungen company [3]. For past more than 25 years, this HELP (Heparin-induced extracorporeal LDL precipitation) is one of the standard methods of extracorporeal LDL-elimination.

A further technological step in the early 1990s was the development of a whole blood LDL-apheresis device, named DALI (Direct Adsorption of Lipids), which removes LDL, VLDL, and Lp(a) directly from whole blood. Because this system does not need any plasma separation step, the handling of the procedure is in some way easier. The adsorber gel in the DALI adsorber columns contain porous Eupergit beads, which allow to pass only plasma but not blood cells to enter inside the hollow beads. The actual lipoprotein adsorbent is polyacrylic acid, covalently bound to the inner membrane of the adsorber beads [4,5].

## Effectiveness and sustainability of LDL-cholesterol lowering by LDL apheresis

At the end of the 1980s, all the above described LDL-apheresis were available for clinical use, so that at this time many data on the effectiveness of LDL-lowering, its consecutive rebound, and the incidence of adverse effects were published.

These data clearly showed that extracorporeal LDL-elimination was superior to all other treatment options available for LDL lowering at that time. For treatment of a defined volume of plasma, the reduction rates for ApoB-carrying lipoproteins ranging from 49 to 75 % based on the value before starting apheresis (Table 1) proved as a target size of all lipid apheresis procedures pushing onto the market [47]. Thus, lipid apheresis was clearly superior to the recommended dietary-drug therapy with resins and fibrates. This did also not change with the first generation of statins.

However, the sustained success of LDL-apheresis depends on the rebound kinetics of lipoproteins and, ultimately, on the treatment intervals—whereby from medical and economic reasons as well as from the patient’s compliance, a weekly interval turned out to be optimal. Lipid-apheresis in patients with severe hypertriglyceridemia is also effective because of rapid lowering of triglyceride-rich lipoprotein particles such as VLDL and chylomicrons. However, due to their rapid rebound they return very quickly to preapheresis values, especially when the patient is anticoagulated with heparin. Therefore, a sustained reduction of these rheologically relevant lipoproteins is only guaranteed if patients are treated in much shorter intervals which can be quite useful in patients with acute pancreatitis due to the hemorheological efficacy of circulating VLDL and chylomicrons [20].

**Fig. 1:**
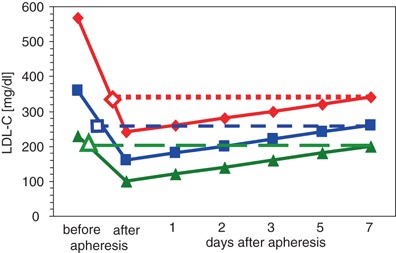
Rebound kinetics of LDL-cholesterol after lipid apheresis**.** The closed symbols are presenting the LDL lowering effect of lipid apheresis in patients with very high (> 500 mg/dl), high (250–499 mg/dl), and low (< 250 g/dl) but still elevated LDL-cholesterol serum concentrations and the reincrease of their individual LDL-cholesterol within two weeks. The LDL serum concentration before the next apheresis (weekly interval provided) is indicated by the open symbols. The sustaining LDL lowering effect one week after the first apheresis corresponds to the difference between the open and the closed symbols

As can be seen from Fig. 1, the long-term effect of LDL lowering also depends on the baseline values. Therefore, the lowering effect in homozygous FH patients with LDL levels clearly above 500 mg/dl is more sustainable than in heterozygous FH patients with LDL baseline values between 250–500 mg/dl [37] if one assumes a roughly comparable resynthesis rate of LDL.

In order to achieve optimal therapeutic target value in patients with hypercholesterolemia, the lipid apheresis has also been used in polygenic FH patients with serum LDL levels below 250 mg/dl not responding to diet and lipid-lowering drugs. As a result of LDL rebound kinetics, the baseline values before apheresis are reached again very quickly (Fig. 1). Therefore the efficiency of treatment is to be questioned under cost-benefit considerations and is actually only justified if a clear clinical benefit can be proved also for these patients.

Shortly after the introduction of lipid apheresis for the treatment of severe FH, the HMG-CoA reductase inhibitors became available for clinical testing in patients who were regularly treated by lipid apheresis but did not reach the therapeutic goal as defined by LDL-target levels. This combination therapy is proved to be an optimal therapeutic approach, particularly when using second and third generation statins, however only in patients with residual ability for LDL-receptor expression. In many patients, especially those with polygenic hypercholesterolemia, the combined treatment modality was so effective that the interval between two apheresis procedures could be extended or in some cases even discontinued [45].

## Clinical efficacy of LDL apheresis

### Case studies (from 1967 onward)

After the introduction of selective LDL-apheresis, the first achieved treatment successes were documented and published as case reports. The patients with homozygous or severe heterozygous familial hypercholesterolemia resistant to therapy, and undergoing regular LDL-apheresis, reported significant improvement of subjective symptoms, such as angina pectoris and exertional dyspnea, after only few weeks of treatment [56].

Xanthomas and xanthelasms regressed markedly within half a year of regular LDL apheresis [33] and showed nearly complete regression after several years of regular treatment.

### Regression studies (1992–1999)

Under the assumption that the effective lowering of LDL by regular apheresis should result in regression of atherosclerotic lesions, several prospective studies were conducted in the early 1990s to demonstrate quantitative changes of coronary lesions [52,49,36,25,19,43,17,30,44].

With a few exceptions, these so-called regression studies were not conducted in a controlled fashion and had the disadvantage of the small number of cases.

The assessment of regression was performed using the so-called quantitative coronary angiography, which should allow a reproducible representation of the vascular lesions under standardized conditions.

One of the two controlled and randomized regression studies is the “Familial Hypercholesterolemia Regression Study” by Thompson and coworkers [49]. The aim of this study was to compare patients who were randomized to drug treatment with statins and cholestyramine or to LDL apheresis at 14-day intervals using dextran sulfate adsorption.

After a mean treatment duration of 2.1 years, the apheresis therapy proved to be superior regarding coronary changes so far. In the group of patients treated by diet and drugs, 21 % of coronary lesions investigated by controlled angiography proved to be progressive whereas in the group of patients additionally treated with apheresis, only 10 % of the lesions showed progression.

The other randomized and controlled study was conducted by a Dutch research group [19]. The angiographically reinvestigated coronary lesions in patients under regular lipid-apheresis were progressive in 43 %. The patients only treated by diet and drugs, however, showed a progression in 52 % of the coronary lesions under investigation.

Japanese colleagues published a historically controlled but not randomized study (L-CAPS). They retrospectively compared patients treated by regular apheresis and patients treated by diet and drugs [25]. In patients treated over a period of two years by a combination of diet, lipid-lowering drugs, and apheresis, 8 % of the coronary lesions were shown to be progressive, while 92 % lesions remained unchanged or were even regressive. The retrospective comparison with drug and diet treated patients showed a progression in 64 % of the examined lesions.

All other so-called regression studies were of longitudinal descriptive nature without comparing to a control group. This means that the initial coronary angiography findings were documented and compared with the final angiography at the end of the study.

The results of these studies have shown similar results independent of the lipid apheresis system used: The progressive coronary stenoses documented by quantitative coronary angiography at the start of regular apheresis therapy remained mostly stable; about one-third of the formerly progressive lesions showed slight regressive changes. However, in 20 % of stenosis, a progression was detected (Table 2).

**Table 2:** LDL-apheresis regression studies. The table summarizes the mean lipid-lowering effects and the segment based analysis of changes in stenosis diameter using quantitative angiography

Study (reference)	Apheresis patients (n)	Reduction of LDL-cholesterol (%)	Quantitative angiography (segment-based analysis; %)		
			NC	R	P
*HELP* [36]	51	62	55	30	15
*LARS* [43]	37	55	49	38	13
*GM* [52]	25	58	64	< 1	36
*FHRS* [49]	20	53	65	25	10
*LAARS* [19]	20	63	45	10	45
	163	53–74	45–64	0–38	10–45

A meta-analysis of different studies of FH patients treated by diet and lipid-lowering drugs alone or combined with lipid-apheresis, followed up by coronary angiography was recently published by Thompson. This analysis showed that the coronary lesions had a noticeable trend to regress in combined treatment of diet, lipid-lowering drugs and apheresis compared to patients without additional apheresis treatment; however, this was not significant at the 5 % level [47].

### Endpoint studies

Neither the extent of reduction in LDL levels nor the proof of regression of atherosclerotic vascular lesions ultimately determines the usefulness or success of any lipid-lowering treatment but only the clinical course of disease and at least the patient’s outcome. Therefore clinical endpoints have to be defined and documented during and after the study period.

Such an outcome trial was conducted by Thompson who treated homozygous twin sisters in the early 1980s [48]: One sibling underwent plasma exchange treatment at regular intervals, the other was treated with conventional diet, fibrates, and cholestyramine. Siblings treated with regular plasma exchange survived their conventional siblings by 5.5 years. Thus, the efficacy of LDL apheresis for patients with homozygous FH was clearly demonstrated.

### Apheresis register (2000–till date)

In order to document the real execution, to ensure the quality of LDL-apheresis and the primary and intermediate medical findings as well as clinical endpoints, thus allowing to make a statement about the success of the therapy, the so-called treatment or patient registers were established. In such registers, adverse effects of treatment, the LDL-lowering efficacy, and other laboratory changes are captured and recorded. Clinical endpoints of therapy such as reinfarction or death are also documented, so that conclusions can be drawn about the event-free survival [40] under therapy. Although a traditional control group is missing, the comparison to other registers or endpoint studies allows insights which might help to finally evaluate the clinical benefit of lipid-apheresis.

The application of HELP simultaneous to its admission to the medical market induced the authorities to give precise orders for monitoring and documentation, similar to a “new drug”. This instruction was basis for the first register in the history of extracorporeal lipid elimination [37].

The detailed analysis of the more than five-year documentation revealed that the event rate based on months of treatment depends inversely on the initial amount of LDL-cholesterol at baseline: HELP treated patients benefited the most, if they had baseline values of more than 250 mg/dl or 500 mg/dl, respectively [37]. It must be therefore seriously discussed whether not only the reduction of LDL levels by 50–60 % is sufficient to reduce the event rate of cardiac end points. This conclusion is based on the known LDL-lowering rates [9,47]: It is hardly conceivable that evaluated HELP register patients could have achieved mean values after single treatment which correspond to target values discussed today.

Currently, in Germany, there are two apheresis registers: The “Quasa” register in Stralsund [8] and the “German Apheresis Register” in Göttingen [34].

At the international level, several apheresis registers have been introduced in the last 10 years [40]. In order to draw valid conclusions from these study registers, the merging of data of comparable quality of documentation is required, because the number of patients treated with the various lipid-lowering devices in the catchment areas of the registers for statistically significant analysis would be too small.

### Pleiotropic effects of LDL apheresis

Besides the desired effect on increased levels of atherogenic lipoproteins, the complex extracorporeal lipid apheresis systems also have more or less desired effects on other plasma proteins, particularly on clotting proteins, preferably on fibrinogen [18,35,14].

Upon contact of the blood and plasma with exogenous materials, inter alia, complement is activated to varying degrees [54]. In the extracorporeal system, the lipids and proteins can be peroxidized but at the same time they can be eliminated by the apheresis procedure [24]. The impact of eliminating cytokines or chemokines, in particular of inflammatory mediators, is only poorly understood. These topics are, at least if one analyzes the latest publications, currently the subject of the research in apheresis [39,6]. Little is known about the effects of eliminating other nonselective, sometimes not even identified plasma components.

The clinical effects observed in patients undergoing regular lipid apheresis can therefore theoretically be interpreted or placed in context by such pleiotropic effects. It is conceivable that the reported subjective and objective clinical improvement under apheresis treatment are the expression of an improved vessel mobility [23,42], improved blood flow [31], and reduced local vascular inflammation combined at the same time with diminished coagulability.

### Indications and cost-benefit ratio

The conditions for financing the health system have changed considerably since the “heydays” of LDL apheresis in the 1980s. The mean costs of a single treatment amount to about 1000 € (these are 25,000–50,000 € per year, dependent on the frequency of the interval), where it is difficult at the present data situation to carry out a plausible cost-benefit analysis.

The indication for LDL apheresis treatment was originally set considering the intervention and target values recommended by the professional medical associations were not achieved by conventional measures such as diet and drug therapy in patients with heterozygous or polygenic FH. Proven drug intolerance was also an indication for apheresis treatment so far as unquestionable atherosclerotic vascular lesions were documented by angiography [12] in patients selected for lipid apheresis. Another prerequisite before approval of any extension of cost funding for continued treatment is a review of the efficacy of LDL lowering. This condition is critical, since until now no available data allow a statement how long patients have to be treated until clinical improvement can be expected. Another unsolved problem is the course of atherosclerosis if maximal therapy is terminated.

The decision to confirm the indication for regular LDL apheresis in Germany is primarily in the hands of the regional apheresis committees. They decide on the basis of a required separate opinion of a “lipidologist” and an independent cardiologist. This setting has only changed little in recent years, especially since the number of patients who require a lipid apheresis treatment is decreasing due to the fact that the current lipid-lowering medication with statins and ezetimibe is highly effective.

The use of apheresis in children with homozygous FH is granted for life in general, because for these patients the cost-benefit ratio is undoubtedly positive.

In the future, new medications and new therapeutic approaches will lead to an even greater reduction of LDL, so that the indication for LDL apheresis will be made even more stringent.

## A look into the future: are there alternatives to LDL-apheresis?

### Liver transplantation

In patients with homozygous familial hypercholesterolemia, the regular LDL apheresis at weekly intervals results in a substantial and sustainable reduction of LDL serum levels and in regression of cutaneous changes such as xanthoma and xanthelasmas. Atherosclerotic stenoses respond to this treatment with a regression, no change and/or slower progression of the documented vascular lesions [27].

However, in the long term, both, costs and the impairment of quality of life by the regular treatments at weekly or biweekly intervals have to be considered. The regular application of LDL apheresis also requires a permanent venous access similar to dialysis patients (arteriovenous fistula), which is associated with a certain risk of thrombosis or sepsis.

The combined heart-liver transplantation or liver transplantation alone is therefore in fact a treatment alternative. While the regularly performed LDL apheresis leads to a mean reduction of LDL-cholesterol by about 50 %, a normalization of LDL-cholesterol is to be expected after successful transplantation [22]. On the other hand, the transplantation cannot be neglected with the risk of acute or delayed organ rejection. The necessary immunosuppressive therapy is associated with risks such as kidney and/or liver failure or the development of a metabolic syndrome. As a result of optimized immunosuppressive therapy used today, the five-year survival rate of liver transplantation in children is at 90 % [15], but in children undergoing regular LDL-apheresis survival is even higher.

### Gene therapy

Even today, the gene therapy is still no real alternative to regular lipid apheresis treatment.

The aim of gene therapy in familial hypercholesterolemia is the overexpression of the LDL receptor by insertion of the receptor-encoding transgene with the help of a suitable vector. So far, the adenoviruses or adeno-associated virus vectors have been found particularly suitable. They infect both resting and dividing cells and remain episomal in the cytoplasm—not in the genome. They are easily to be manipulated at the molecular level so that they can be produced at any high titers [46]. However, they have a high immunogenicity, i.e., a cellular and humoral immune response against the foreign protein leads to the elimination of hepatocytes infected by the vector.

So far, there is only one trial for the treatment of homozygous FH in humans. In five patients, partial resections of the liver were performed, the hepatocytes were isolated, cultured, and then infected with one of the LDL receptor gene-encoding retroviruses. The liver cells were then reinfused into the portal vein. In three of the five patients in this pilot study, this treatment lowered the LDL cholesterol by 6–25 % [13].

An updated overview of the now 18-year tried approaches to establish a sustainably applicable gene therapy is given in a recent review by Al-Allaf [2]. A qualification in the significance of lipid apheresis cannot be derived from this paper, however, not in the near future.

### New lipid-lowering drugs

In the future, further lipid-lowering agents will be available in the market, which will achieve a sufficient reduction in LDL-cholesterol in many patients [29]. In particular is the development of antisense oligomers (Mipomersen) to name. The first experience with this kind of new drugs indicates a sustained reduction in LDL in homozygous FH (− 47 %) and also a significantly lower Lp(a) (− 31 %) relative to the baseline without treatment. The medication is not entirely free of side effects: With few exceptions, the patients reported local reactions at the injection site and flu-like symptoms [16].

The role of LDL-apheresis will lose its importance in future as a result of this development and is expected to actually be confined to cases with FH, which have no therapeutic alternatives because of not sufficient LDL-lowering because of drug incompatibility reasons or ineffectiveness of the new lipid-lowering drugs.

How each of the discussed pleiotropic effects, in particular the rheological effectiveness for patients compared to drug therapy, represents an advantage, must remain open to the absence of related studies.

Even the debate about the use of LDL-apheresis as a last resort in the isolated increase of Lp(a) is offset by the future availability of drugs that effectively reduce Lp(a), this in its meaning still controversial discussed lipoprotein [16]. However, the anti-atherogenic role of Lp(a)-reduction can be demonstrated only by a therapy that is exclusively eliminating Lp(a). Therefore the specific Lp(a)-immunoadsorption might be the remaining extracorporeal lipid-apheresis method in the next decade.

#### Conflict of interest

The authors declare that there is no conflict of interest.

#### Acknowledgment

This article is part of a supplement sponsored by an unrestricted educational grant from B. Braun and Fresenius Medical Care.
